# Responsibilities and capabilities of health engagement professionals (HEPs): Perspectives from HEPs and health consumers in Australia

**DOI:** 10.1111/hex.13155

**Published:** 2020-11-11

**Authors:** Lisa Tam, Kara Burns, Katherine Barnes

**Affiliations:** ^1^ School of Advertising, Marketing and Public Relations Queensland University of Technology Brisbane Qld Australia; ^2^ The Hopkins Centre Menzies Health Institute Queensland Griffith University Logan Qld Australia; ^3^ Queensland University of Technology Brisbane Qld Australia

**Keywords:** Australia, co‐design, consumer engagement, health consumers, health engagement professionals, patient involvement

## Abstract

**Background:**

In Australia, the National Safety and Quality Health Service Standards (2012) stipulates that partnering with health consumers to improve health‐care experiences is one of the criteria health‐care organizations are assessed and accredited against. This standard has given rise to a role: health engagement professionals (HEPs). While there are no standard requirements for recruitment into this role, this study contributes to much needed research into understanding their responsibilities and capabilities, and their contributions to engagement outcomes.

**Methods:**

Using a qualitative, interpretive approach, 16 HEPs and 15 health consumer representatives (who have experiences of interacting with HEPs) participated in an in‐depth phone interview in December 2019. We explored (a) the purposes of the role, (b) the responsibilities and work activities and (c) the capabilities required to carry out the responsibilities.

**Results:**

Health engagement professionals are specialists in designing engagement mechanisms for health‐care organizations to co‐design health services with health consumers. They facilitate partnerships between health‐care organizations and health consumers. They play significant roles in listening to, facilitating understanding amongst different stakeholder groups (eg hospital management, health‐care workers and health consumers) and navigating the bureaucratic structures to influence outcomes. Four major responsibilities (advocacy, education, facilitation and administration) and four categories of capabilities (relational, communication, professional and personal) were identified.

**Conclusion:**

A list of job responsibilities and desired capabilities of HEPs is provided to help health‐care organizations better understand the requirements for the role. This would help them decide how applicants to these roles would meet the requirements (eg experience of navigating bureaucratic systems).

## 1 INTRODUCTION

The primary aims of the National Health Service and Safety Quality Standards^1^ in Australia are to protect health consumers from harm and to improve the quality of health service provision through criteria established in consultation with a wide range of stakeholders. Health‐care services can use these standards as a part of internal quality assurance and must use them to meet a minimum level of performance when used as part of an external accreditation process. The current and immediate past standards request that health‐care facilities ‘partner with consumers’ to improve health‐care experiences. To meet this standard, a new type of health‐care management professional, referred to as health engagement professionals (HEPs) herein, is emerging alongside existing patient advocates and consumer complaints officers.

Consumer engagement (also known as patient engagement or patient involvement) is essential to improving the quality, safety and delivery of health care.^2^ Current research has generally defined consumer engagement as engaging health consumers in ‘designing or improving health services through activities such as completing surveys about their care experiences or serving as advisors or members of governance or quality improvement committees'.^3^ This practice has evolved in recent years as more research has been conducted to examine consumer partnership in health‐care and system planning and decision making. For example, a Canadian study explored motivational factors underlying patient engagement which guides health‐care organizations to develop initiatives to better create meaningful engagement opportunities for health consumers.^2^ Another Canadian study developed an evaluation tool that identifies the core principles for quality public and patient engagement.^4^ In Australia, consumer engagement is a mandatory requirement of hospital accreditation and health consumers are typically referred to as individuals with lived experiences of health‐care services.^5^ The principle of co‐design is applied to invite health consumers to involve in the planning, development and evaluation of health services.^5^ Co‐design is a principle within consumer engagement that stipulates that individuals with a shared interest in designing and improving health services should be involved in consumer engagement.^2^ To ensure that engagement activities contribute to the outcome of improving health services, health service organizations are advised to provide health consumers with training and resources.^6^ It is recommended that engagement moves beyond the provision of information, through to active engagement and empowerment of consumers to co‐design health‐care services.^5^, ^7^

Because consumer engagement is mandatory for hospital accreditation, roles specializing in consumer engagement have arisen. This study refers to them as health engagement professionals (HEPs). In the United Kingdom, they are known as patient and public involvement facilitators (PPIF) whose primary responsibility is to involve patients in service improvement projects.^8^ In Australia, HEPs are specialists in developing engagement frameworks and techniques for health service organizations to meet the requirement of the *National Health Service and Safety Quality Standards'* ^1^ *Partnering with Consumers Standard*. According to the Standard, health service organizations should work with health consumers ‘as partners in planning, design, delivery, measurement and evaluation of systems and services’ and with patients as ‘partners in their own care, to the extent that they choose’.^9^ Partnerships take place at the individual, service and organization levels to facilitate person‐centred care such that care prioritizes the relationship between patients and clinicians for the best health outcomes. But successful consumer partnerships are not without challenges—it requires health services and consumers to redefine their roles and responsibilities, increases costs and conflicts between health consumers and professionals, leads to slower progress in change and could result in tokenism.^6^, ^8^, ^10^, ^11^

Existing research has examined the roles of health consumers (also known as patient advisors in the United States and Canada) who are patients who convene on a regular basis to improve service delivery in patient advisory councils (also known as consumer representative groups in Australia).^11^, ^12^ The role of HEPs has been researched in the United Kingdom where they are known as patient and public involvement facilitators (PPIF) who play the roles of gatekeepers (ie determining which patients get access to patient and public involvement (PPI) activities), mediator (ie facilitating conversations between staff and patients in committees) and negotiator (ie encouraging health‐care professionals to involve patients in projects and implement changes).^8^ Although the study^8^ has identified some of their responsibilities, the authors recommend that further research be conducted to provide clear definitions of their specific roles and the nature of their responsibilities. Thus, this study is conducted to examine the roles of health engagement professionals (HEPs) who are in paid positions and are embedded within health service organizations in Australia. Their roles are critical for several reasons. First, consumer partnership is founded on the idea that ‘healthcare is a human, relationship‐based activity’.^13^ HEPs are the boundary spanners between health service organizations and health consumers who facilitate that activity. Second, the purpose of the relationship is to create a ‘supportive ecosystem’ whereby consumer partnerships can lead to the co‐production of health services.^13^ In addition to designing the activity, HEPs also need to contribute to the creation and maintenance of that supportive ecosystem. Third, although there is ample empirical evidence on consumer engagement and how it should be conducted, the roles of HEPs are not well‐understood. Because of this, they could be constrained by limited training and mentoring opportunities and inadequate financial and physical resources.^8^ Research on community engagement professionals (CEPs) has identified the need to understand their knowledge, skills and abilities in order to ensure that they can facilitate partnerships that contribute to the outcome of engagement.^14^ To date, there is limited research on HEPs and this omission in the literature is striking given they set up and implement the interactions for consumer engagement ‘on the ground’.^15^ Thus, this study examines an overarching research question: What are the responsibilities and capabilities of health engagement professionals (HEPs)?

## 2 METHODS

To address the research question, we (a) conducted a review of responsibilities of HEPs and CEPs; (b) conducted a literature review of research on capabilities of health‐care professionals; (c) identified categories of capabilities and developed an interview guide; (d) obtained ethics approval from the University; (e) recruited participants and collected the data using interviews; and (f) analysed the data based on the process of thematic analysis. Prior to data collection, this study was approved by the University's ethics approval committee in July 2019 (reference number: 1900000303).

### 2.1 Phase I literature review

We began formulating this study by browsing the job advertisements looking to recruit HEPs in Australia from which we found a diversity of job titles and no defined responsibilities and capabilities. Since we could only identify one study on HEPs,^8^ we conducted a literature review of community engagement professionals (CEPs) on their job responsibilities instead. Our literature review identified several categories of responsibilities including the need to ensure diversity and equity in their work,^16^ to advance engagement practice and to influence administrators,^16^ to act as intermediary and project manager and to ensure project development in terms of complying with laws and regulations,^15^ to advocate for system and process change^17^ and to participate in and lead the strategic planning process as ‘institutional change leaders’.^18^ The ‘relational work’ that CEPs are engaged in requires them to be self‐aware, conscious of power relations and knowledgeable of resources and opportunities in order to build trusting relationships and partnerships.^19^

Although research on CEPs has helped to identify some of the responsibilities that are also applicable to HEPs in the health context, such research has not addressed the question of the capabilities which are needed to carry out the work. As such, we also completed literature review on essential capabilities in the context of health. Although there is no research dedicated to the capabilities of HEPs, we identified four categories of capabilities that applied to other health professionals. First, as the relationship between patients and health professionals is characterized by asymmetry in power and authority, *relational capabilities* are amongst the most critical key attributes that contribute to improving interactions to positively affect health perceptions and outcomes. Specifically, these relational capabilities include not sounding authoritative and dominant^20^ and the demonstration of empathy,^21^, ^22^ cultural sensitivity and respect.^23^ Second, *communication capabilities*, including active listening,^24^ assertiveness,^25^ non‐verbal communication,^26^ showing a genuine interest in patients by encouraging questions^27^ and providing timely, accurate and frequent communication,^28^ are identified as some of the key attributes. Third, *personal capabilities* or qualities, including emotional intelligence,^29^ being open and honest^30^ and self‐awareness,^31^ are amongst the key attributes identified. Last and above all, *professional capabilities* (ie skills which are essential to fulfilling the job responsibilities) include leadership,^32^ educator,^33^ advocacy^34^ and being able to navigate conflicts and barriers.^35^

### 2.2 Phase II development of interview guide

Upon completion of literature review, an interview guide was constructed. The guide comprises questions including the key responsibilities of the role of HEPs, the activities that fall under these key responsibilities and the four categories of capabilities. The four categories of capabilities are defined as follows in the context of this study. For HEPs, the category of *relational capabilities* refers to capabilities essential to the management of relationships with health staff and health consumers such as empathy and respect. The category of *communication capabilities* is defined as capabilities essential for the receipt and transmission of information. The category of *professional capabilities* is defined as those essential for meeting the professional requirements of the role such as advocacy and education. Lastly, the category of *personal capabilities* refers to individual qualities which are complementary to the other three categories of capabilities such as honesty. Some of the questions asked included ‘What do you think is the main purpose of the health engagement advisor role?’, ‘What are the key responsibilities of the health engagement advisor roles?’, ‘Can you give me some examples of the kinds of activities health engagement advisors do?’ and ‘What are the relational/communication/personal/professional capabilities do you think are important in the health engagement advisor role?’.

### 2.3 Phase III data collection

After obtaining approval from the University's ethics committee to proceed with data collection, our research study was advertised on Health Consumers Queensland's (HCQ) newsletters in November 2019. HCQ is a non‐profit peak organization that represents the interests of health consumers and carers in the Australian state of Queensland.^36^ It provides resources and training and skills development opportunities for health consumers, carers and staff to maximize consumer representation at all levels of the health system. Sixteen health consumers and carers and fifteen HEPs were recruited to participate in phone interviews which were audio‐recorded in December 2019. Data collection ended when data saturation was achieved. The length of the interviews ranged from approximately 25 to 65 minutes. Participants were offered an AUD$30 online grocery gift card for their time. Of the 31 participants, five were male and 26 were female. Two of the health consumers were from culturally and linguistically diverse backgrounds.

### 2.4 Phase IV data analysis

The audio‐recorded interviews were transcribed first automatically using an automated transcription service, Trint, and were then manually checked and revised by the first two authors of this study. The authors then followed the process of thematic analysis to analyse the interview transcripts.^37^ Thematic analysis is useful for identifying, analysing and reporting themes (or patterns) within data during the process of which researchers play an active role in discovering themes, selecting which themes are of interest and choosing certain themes to be reported.^37^ The process of thematic analysis allows the researchers to code and analyse the data based on what they want to know. The first two authors first read through all the data after which they discussed their preliminary findings. They then proceeded with open coding and discussed their definitions and categorizations of the codes identified. Upon reaching an agreement, they proceeded with axial coding to group the codes into categories. Each HEP participant was de‐identified with an anonymous code ranging from 1A to 15A with ‘A’ denoting ‘advisor’, and each health consumer representative participant was de‐identified with an anonymous code ranging from 1C to 16C with ‘C’ denoting ‘consumer/carer’.

## 3 RESULTS

The findings identified the specialized role of HEPs as specialists in designing engagement mechanisms to facilitate consumer partnerships, defined as the bridge between hospital management/staff and health consumers/carers. Health consumers and carers are invited to convey their voices in the research, planning, design, delivery and evaluation of health services, and HEPs advocate for them as a group when they are not able to advocate for themselves. HEPs develop systems to enable health consumers/carers to co‐design health services which reflect a better understanding of their needs and result in better care.

### 3.1 Responsibilities and activities of HEPs

The responsibilities of HEPs are coded into four categories: advocacy, education, facilitation and administration. These responsibilities are outlined in Table 1.

**TABLE 1:** Four categories of responsibilities and related activities

**Advocacy:** to advocate on behalf of the health consumers and to encourage consumers to be heard
Promote consumer inputs in the system
Create a receptive environment to influence change and influence culture to make sure it is person‐centred
Advise and give policy recommendations
Work with hospital boards and management
**Education:** to educate on consumer engagement practices
Provide health consumers with information such as patients' rights, engagement opportunities and feedback
Attend community and consumer forums to promote engagement opportunities
Interpret information for health consumers
Provide feedback from consumers to health staff and management
Provide training opportunities (eg workshops) for health consumers and health staff to be involved in consumer partnerships
Build capacity of health staff to work effectively with health consumers
**Facilitation:** to involve users of the health system to make and influence decisions in the research, planning, implementation and evaluation of health services
Enable health consumers to see health staff's point of view and vice versa
Address concerns from and meet with health consumers and health staff and resolve disputes
Organize, coordinate and facilitate engagement activities appropriate for different audience and for different purposes such as survey, committees and workshops
Manage consumer networks to decide who sit on different committees and are involved in different projects
Ensure a diversity of voices are heard and facilitate understanding
Maintain relationships with stakeholders
**Administration:** to perform the administrative tasks necessary for their roles as be a bridge/intermediary/liaison amongst health consumers, health staff and top management
Develop (a) policies and processes that enable consumer partnerships, (b) tools and resources for training health staff and consumers and (c) frameworks of engagement
Conduct project management including organizing paperwork, setting up systems and working with IT
Keep a register to ensure accreditation requirements and key performance indicators (KPIs) are met
Ensure engagement is aligned with strategic goals

#### 3.1.1 Advocacy

The responsibility of advocacy comprises activities including (a) the promotion of consumer inputs in the health system, (b) creating a receptive environment to influence change and influence culture to make sure it is person‐centred, (c) reviewing and providing policy and procedure recommendations, and (d) working with hospital boards and management. Advocacy is identified as a primary responsibility as HEPs are internal advocates who bring about changes in the system. One HEP [6A] emphasized that the role ought to ‘help embed [engagement] into practice at all levels’ and further added that ‘they need to be able to fill the capacity of others in the system to do it and to see the value of doing that.’ Another HEP [10A] emphasized that ‘consumer engagement needs to be in every single person's role’ and that her role ‘is to help the organization I work for build consumer engagement into all the systems’. One health consumer [8C] also noted that HEPs could be ‘advocating on behalf of the person who is incapable of advocating on their own behalf’, ‘advocating on behalf of a group of people who have special needs’ or ‘simply advocates or representatives on a group advising on a range of health issues from communications to health care to building design to delivery of health services in the home to promote care’. Moreover, such advocacy is not limited to health service organizations but should be extended to ‘the state to the Commonwealth government, so through the process to gain additional services and to promote the best outcome for the person with the health issue’ [8C].

#### 3.1.2 Education

The responsibility of education comprises activities including (a) educating health consumers about patients' rights, engagement opportunities and feedback, (b) interpreting information for health consumers, (c) providing feedback from consumers to health staff and management, and (d) providing training opportunities for health consumers/carers and staff to be involved in consumer partnerships. One HEP [7A] commented that one of her responsibilities is to help health staff within the health service to see ‘what these roles are capable of, because I think a lot of them see these roles as trying to achieve our accreditation and national standards as compliance’. Another HEP [9A] noted that in order to demonstrate best practice in consumer engagement using engagement techniques and methodology, they also have the responsibility to develop tools and templates for the organization to use to carry out engagement and to educate health staff what it is and how to use it. They also develop resources for training HEPs and health consumers/carers to participate in formalized engagement opportunities. This role requires collection, interpretation and translation of information and knowledge between all parties, in a way that respects all stakeholders.

#### 3.1.3 Facilitation

The responsibility of facilitation comprises activities including (a) addressing concerns from health staff and health consumers, (b) enabling them to see the other party's perspectives and facilitating understanding between and amongst them, (c) organizing and coordinating engagement activities between and amongst health consumers/carers, and (d) maintaining relationships with all stakeholders. For health consumers, they interact with HEPs most when HEPs facilitate group meetings amongst health consumer representatives. A health consumer [1C] provided an example of how a HEP facilitated a meeting that sought consumers' feedback on posters that were designed to be used in emergency departments to make sure diverse members in the group could all understand the wording. A HEP [2A] also provided an example of conducting engagement about how to make waiting areas more person‐centred. Another HEP [4A] commented that facilitation of diverse consumer voices is important. She shared her experiences of when facilitation of formalized consumer groups could fail:

We've done a couple of reviews of consumer groups where they have really fallen down. And actually, it [is our] responsibility ensure that we're doing no harm. And sometimes I think these groups can be harmful. They can make people feel they are losing confidence in their voice or they feel they don't know where their input is going. So it's certain that it takes people a bit of time to learn how to operate within those types of structures. So I think we have the responsibility of making sure that people do it in a way that speaks for everyone and not disempowering and not, you know, if there's conflicts within the group.

#### 3.1.4 Administration

The responsibility of administration comprises activities at the organizational level including (a) developing policies and processes that facilitate consumer partnerships, (b) conducting project management such as setting up IT systems and ensuring that health consumer representatives are paid for their involvement, (c) keeping a register to ensure that accreditation requirements are met and the alignment of engagement activities with organizational goals, and (d) other miscellaneous administrative tasks such as creating forms for health consumers to sign up to become representatives. One HEP [6A] provided the example of developing a remuneration statement for health consumer representatives to ensure that they would not be out‐of‐pocket for their work and are properly reimbursed. As for health consumers, they see the provision of feedback on consumer inputs and the recruitment of appropriate health consumer representatives to be key administrative activities. One of them [13C] noted that HEPs need to administer tasks like making sure ‘that people turn up on time, are fed and watered and have their parking paid for’.

### 3.2 Capabilities

The four categories of capabilities are outlined in Table 2.

**TABLE 2:** Four categories of capabilities

**Relational capabilities:** essential to the management of relationships with health staff and health consumers
Compassion and/or empathy
Negotiator, translator and interpreter
Culturally aware (‘inclusive’ and ‘respectful’)
Able to engage with diverse populations and build interpersonal relationships
Understanding group dynamics and power differentials
Capacity building for others
**Communication capabilities:** essential for the receipt and transmission of information
Active listening and understanding
Knowing when to talk
Able to communicate both face‐to‐face and in written communication
Able to manage difficult communication
**Professional capabilities:** essential for meeting the professional requirements of the role such as advocacy and education
Able to influence improvement in a system characterized by strong governance
Able to navigate the bureaucratic structure
Policy literate; process literate; health literate; technology literate
Researcher; educator; facilitator
Strategic thinker
Innovation (‘willing to experiment’)
**Personal capabilities:** individual qualities which are essential to complementing the other three categories of capabilities
Self‐awareness to accept others' views (‘non‐judgemental’)
Persistence/determination as the system takes a long time to change
Person‐centred
Authentic, flexible, honest, nice, genuine and patient
Friendly and approachable

#### 3.2.1 Relational capabilities

To fulfil the responsibilities (and activities), the capability to build and maintain relationships with health staff/management and health is amongst the most critical capability. The words ‘relational’ and ‘relationship’ were the most frequently mentioned in the interviews. One HEP [7A] describes health engagement as a ‘relational field’ and that she would look for potential applicants who had experiences in working with people and groups of people for the roles. Another HEP [4A] emphasized that the individuals recruited into these roles should have ‘extensive relationships’ and to be ‘trusted in the community’ and a ‘very grassroots kind of person’. The themes found for relational capabilities include (a) compassion/or empathy, (b) cultural sensitivity (in terms of being able to engage with diverse populations), (c) understanding of group dynamics and power differentials, and (d) being able to negotiate, translate and interpret meanings from different individuals and for different individuals. While multiple health consumers have emphasized the importance of empathy, there are mixed findings from HEPs about the importance of empathy. One HEP [1A] commented: ‘The argument around compassion, rather, is empathy. You know, empathy can lead to burn‐out because you're taking on board too much of the emotional load as opposed to the concept of compassion, which I guess, is about caring’. Likewise, another HEP [4A] also noted that HEPs ‘have to know their boundaries’ because ‘that can be quite emotionally difficult at times’. It is noteworthy that HEPs, being strongly committed to the process of consumer engagement, have expressed distress when feeling that they have failed the health consumers. A HEP [4A] further added that ‘It's the role of being that intermediary like one foot across two camps in a way. And that is difficult. And if we don't recognize that, then we're going to burn these people out just like we burn other roles out’. But from a health consumer's perspective [2C], it was shared that it was necessary ‘to have some involvement at an emotional level with patients and look after them when things happen’. On a positive note, health consumer representatives appreciated HEPs’ cultural sensitivity and power differentials. One health consumer representative [1C], who is from a culturally and linguistically diverse background, appreciated that HEPs ensured that she was given an opportunity to talk in the group when others dominated the discussion.

#### 3.2.2 Communication capabilities

While the importance of communication capabilities is more or less a cliché, there are specific communication capabilities essential to the role of HEPs. The themes identified include (a) active listening and understanding, (b) knowing when to talk, (c) ability to communicate in both face‐to‐face and in written communication, and (d) managing difficult communication. HEPs and health consumers/carers emphasized that consumer engagement must not be a tokenistic, tick‐box activity and the communication of HEPs is critical to making health consumers/carers to feel valued. HEPs are their go‐to person when health consumers have issues [4C]. A health consumer [3C] shared the importance of ‘closing the loop’ by informing health consumers of how changes are being embedded into the systems. Likewise, A HEP [13A] also notes that consumers should be able to get feedback ‘to understand that their time and effort and their feedback has been taken onboard and it has made a difference’. Another HEP [8A] noted that when consumers are not given feedback on what has been achieved and what has not been achieved, the communication will break down as trust will be negatively affected. Moreover, people ‘might be consulted over and over again on the same subjects, but nothing ever changes’. Another health consumer [4C] noted that non‐verbal communication such as body language, posture, eye contact and hand movements is helpful in increasing understanding. Moreover, it is important that they use ‘simple language, not clinical language’. Meanwhile, one health consumer [1C] noted that the frequency is not important but ‘the right amount’; HEPs need to follow through by providing materials to read before the meetings and providing timely feedback after the meetings.

#### 3.2.3 Professional capabilities

Professional capabilities, defined as those essential to fulfilling the job responsibilities, include themes such as (a) advocating for improvement in the system, (b) being health literate, policy literate and process literate, (c) being a leader, strategic thinker, researcher, educator and facilitator, and (d) willing to experiment and to bring about innovation. In terms of advocacy, HEPs' ability to influence, including influencing the governance, is important [9A]. By having relationships with both health staff and health consumers, their advocacy role becomes more powerful [17A]. On the other hand, a HEP [8A] stressed that HEPs ‘should be advocates for improvement in the system’ but not ‘an advocate for an individual consumer’ because there are other roles for that. Nevertheless, their ability to influence could be dependent upon how they are institutionalized into the system [1A, 8A]. Some of them could be housed in the communication departments where their primary role could be information dissemination. A health consumer [6C] commented that the facilitative role of HEPs requires them to ‘be on both sides of the fence’ that they might sometimes be on the sides of health consumers and sometimes on the sides of health staff/management. A HEP [1A] described that ‘there can be a little bit of tension between consumers and health professionals. Sometimes I feel like an intermediary who has to negotiate, I suppose, between the two parties’. They should be able to get one party to see the other party's point of view. They also need to make sure that ‘no one dominates, that everyone has an opportunity to speak in equal measure’ [3C] As for innovation, a HEP [8A] shared that her organization implemented a consumer community online and a web platform so that consumers could connect with them on a regular basis and receive regular updates on opportunities to be engaged. This capability is particularly important when some consumer groups can be ‘staid’ and ‘siloed’ such that they are no longer challenging the system [4A]. Thus, innovative methods of engagement often need to be brought in to spark creativity.

#### 3.2.4 Personal capabilities

Although personal capabilities could be considered complementary, we propose that personal capabilities are critical to achieving success in these other three categories and should also be included as essential foundation, not complementary. Personal capabilities reflected some personal beliefs which supported the other three categories. The themes identified include (a) self‐awareness to be able to accept others' views (being non‐judgemental), (b) persistence and determination as the bureaucratic system takes a long time to influence and (c) being friendly, approachable, authentic, flexible, honest, genuine and patient. A HEP [6A] shared that being self‐aware of one's own privilege and worldview helped HEPs accept others' worldview. A health consumer [3C] shared a similar view because self‐awareness is helpful for ‘not coming with pre‐conceived ideas’. By not being judgemental, they could be ‘more welcoming, more inclusive’ [6A]. Having to work up the bureaucracy as a formal process requires patience and persistence because ‘making change is very, very slow’ [1A]. It is important to take some risks by experimenting new things in order to build up the change [7A]. Meanwhile, HEPs should make health consumers feel that ‘no one is of authority’ [3C] and demonstrate emotional intelligence [1A, 6A, 3C] in terms of being sensitive to the differences amongst people in the room and bringing bad news to people. They need to be willing to hear consumers' voices without trying to change it (even if it is unpleasant) [2C].

## 4 DISCUSSION

This study has identified a list of responsibilities (and activities under reach responsibility) and four categories of capabilities for the roles of HEPs. Table 3 shows a summary of noteworthy findings.

**TABLE 3:** Summary table of noteworthy findings

Loyalty to whom?
HEPs stressed that they were not advocates for individual consumers; they are advocates for improvement in the systems. On the other hand, health consumers emphasized that HEPs' primary responsibility was to advocate for individual consumers.
Tension could arise as HEPs are responsible for choosing a selected group of individuals to serve as health consumer representatives to represent the collective voices of all health consumers in the process of consumer engagement. It is important to ensure that the ‘selected’ voices can represent the collective in improving the systems.
HEPs ought to believe that system‐level change is possible and be committed to follow through on how the voices of health consumers are incorporated into the systems.
The roles of HEPs could be embedded into health service organizations differently. Some of them could have other responsibilities such as handling patient complaints. This could affect the roles of HEPs in fulfilling their responsibility as advocates for improving the system.
Empathy vs compassion?
Some health consumers conveyed that HEPs should have ‘lived experiences’ like the patients themselves so that they could empathize with them. But HEPs highlighted that they needed compassion and boundaries because they were not trained in counselling.
There is a need to re‐examine the attribute of empathy in health‐care professionals and distinguish it from compassion.
Can HEPs be trained?
The job descriptions for HEP roles do not often have predefined required and desired qualifications and capabilities.
Some of the capabilities identified in this study including the relational, communication and personal capabilities may come *with* the person and cannot be acquired by training; an example is one's commitment and persistence to navigate through the bureaucratic systems.
Some professional qualities such as health literacy, process literacy and policy literacy can be acquired through training.

### 4.1 Loyalty to whom?

HEPs are not advocates for individual consumers (even though health consumers have emphasized this responsibility), but they are advocates for changes in the systems that support health consumers as a group. Although HEPs felt that they were advocates for improvement in the system, not advocates for individual consumers, tensions could arise as HEPs were also the ones responsible for hand‐picking a selected group of health consumer representatives to represent the collective voices of all health consumers in the process of consumer engagement. Thus, there could be an ongoing tension between meeting individual and collective needs. And health‐care organizations need to provide services based on the needs of the majority, rather than individual needs. Hence, to advocate for changes in the systems, HEPs ought to ensure that the health consumer representatives involved in the process could represent the voices of the majority. Also, HEPs ought to believe that system‐level change is possible and be committed to follow through on how the voices of health consumers are incorporated into the systems. Despite this, their roles could be embedded into health service organizations differently. For example, some of them could also wear the hat of a patient complaint advisor or are embedded into a communication department. They could have different titles such as engagement advisors and patient advisors.^11^ Future research should provide clarity on how being embedded into a different department or having other responsibilities could affect the roles of HEPs in fulfilling their responsibility as advocates. There is also a need to examine the distinction amongst these roles so that the roles of HEPs can be clarified. Moreover, some consumers have identified that HEPs are ‘supportive’ roles who facilitate the meetings and provide administrative support and may not have real power to influence the systems.

### 4.2 Empathy vs compassion?

HEPs and health consumers differ in their views on the importance of empathy as a relational capability. Some health consumers have conveyed that HEPs should have ‘lived experiences’ as patients themselves so that they could empathize with them. One health consumer [4C] emphasized that empathy, not sympathy, was needed, and another health consumer [5C] highlighted the value of empathy in helping HEPs understand health consumers and move the relationships forward. But as the data conveyed, HEPs highlighted that compassion and boundaries are needed; they are especially crucial because they were not trained in counselling. Existing research on compassion in health care has defined compassion as having ‘a deep awareness of suffering of another, coupled with a wish to relieve it’^38^ and that a compassionate person ‘recognizes and acknowledges the plight of another at an emotional level’.^39^ But compassion can result in compassion fatigue, resulting in burnout, misjudgements and errors.^40^ This finding also points to the need of re‐examining the attribute of empathy that existing research has found to be important in health‐care professionals.^22^, ^41^, ^42^ Although empathy is desirable in the eyes of health consumers, distinctions should be made between empathy and compassion and future research should look into the differences between health‐care professionals' portrayal of empathy and health consumers' experiences of their empathy.

### 4.3 Can HEPs be trained?

Because job advertisements for HEP roles do not often have predefined required and desired qualifications and capabilities, the question of whether HEPs can be trained remains unanswered. Although the position descriptions may show a list of job responsibilities and associated activities, some of the capabilities identified in this study including the relational, communication and personal capabilities may come *with* the person and cannot be acquired by training. For example, self‐awareness and persistence to commit to consumer partnerships and to navigate through the bureaucratic structure are personal qualities that are likely to have been developed in individuals over time. However, professional qualities such as health literacy, process literacy and policy literacy could be acquired through training. Acknowledging that it could be challenging for health service organizations to recruit HEPs with all the capabilities, future research could examine how some of these capabilities might be prioritized over others.

## 5 CONCLUSION

This study has contributed to the understanding of HEPs as intermediaries between health service organizations and health consumers. In their roles, the most important yet the most difficult activity in partnering with consumers is often the shift of control from the health‐care professionals to the health consumers to create a ‘balanced’ partnership.^10^ To date, consumer engagement is well‐understood in the health setting but the roles of HEPs in this practice require further research. This study has identified their job responsibilities and associated activities and the capabilities required or desirable for the roles. Nevertheless, many still consider consumer engagement to be a ‘tick‐the‐box’ obligation carried out to meet accreditation requirements, consistent with the findings from a study in the United Kingdom.^8^ Furthermore, more education regarding the practice of consumer engagement and HEPs within the health‐care organization and in the communities is still needed. Without such understanding, the ability of HEPs to influence and bring about changes in the system could be limited.

## 6 STUDY LIMITATIONS AND FUTURE RESEARCH

This study has several limitations. First, this study was conducted in Australia and the findings might not be generalizable to other countries. Future research should explore the roles of HEPs in other countries. Second, although interviews were conducted with both HEPs and health consumers to explore their understanding of consumer engagement practice and the roles of HEPs, most health consumers' experiences of HEPs are limited to certain activities such as committee meetings and email communication. Future research should also explore health‐care professionals' and management's experiences of the practice of consumer engagement and the roles of HEPs. Third, some of the responsibilities and capabilities identified are overlapping and are not mutually exclusive. Future research may use the findings from this study as a basis to develop a quantitative scale to better measure the categories of responsibilities and capabilities and their effects on the outcomes of consumer engagement. Fourth, this study did not ask HEPs or health consumers to describe the outcomes of their involvement in and contributions to consumer engagement. Future research should consider examining the impact of their work and how the performance of HEPs is evaluated by their employers. Lastly, the HEPs interviewed in this study were primarily responsible for consumer engagement but they could be embedded in the organizational structures differently and could have other responsibilities (eg in the departments primarily responsible for handling communication or patient complaints). Future research should further examine how this could affect their access to resources or support within the organizations.

## CONFLICTS OF INTEREST

The authors whose names are listed in this study certify that they have no affiliations or involvement in any organization or entity with any financial or non‐financial interest in the subject matter or materials discussed in this manuscript.

## PATIENT OR PUBLIC CONTRIBUTION

Sixteen health consumer representatives and fifteen health engagement professionals (HEPs) participated in this study as interview participants.

## Data Availability

Research data are not shared.
